# Antioxidant efficiency of lycopene on oxidative stress - induced damage in bovine spermatozoa

**DOI:** 10.1186/s40104-016-0113-9

**Published:** 2016-09-06

**Authors:** Eva Tvrdá, Anton Kováčik, Eva Tušimová, Dušan Paál, Alica Mackovich, Jakhongir Alimov, Norbert Lukáč

**Affiliations:** 1 Department of Animal Physiology, Faculty of Biotechnology and Food Sciences, Slovak University of Agriculture in Nitra, Tr. A. Hlinku 2, Nitra, 94976 Slovakia; 2 AgroBioTech Research Centre, Slovak University of Agriculture in Nitra, Tr. A. Hlinku 2, Nitra, 94976 Slovakia; 3 Department of Botany and Genetics, Faculty of Natural Sciences, Constantine the Philosopher University in Nitra, Nábrežie mládeže 91, Nitra, 94974 Slovakia; 4 Department of General Biology, Faculty of Natural Sciences, Gulistan State University, 4th Microrayon, Guliston, 120100 Syrdarya Uzbekistan

**Keywords:** Antioxidants, Bulls, Ferrous ascorbate, Lycopene, Oxidative stress, Spermatozoa

## Abstract

**Background:**

Lycopene (LYC) is a natural carotenoid with powerful reactive oxygen species (ROS) scavenging activities. The aim of this study was to investigate if lycopene has the ability to reverse ROS-mediated alterations to the motility, viability and intracellular antioxidant profile of bovine spermatozoa subjected to ferrous ascorbate (FeAA). Spermatozoa were washed out of fresh bovine semen, suspended in 2.9 % sodium citrate and subjected to LYC treatment (0.25, 0.5, 1 or 2 mmol/L) in the presence or absence of FeAA (150 μmol/L FeSO_4_ and 750 μmol/L ascorbic acid) during a 6 h *in vitro* culture. Spermatozoa motion characteristics were assessed using the SpermVision™ computer-aided sperm analysis (CASA) system. Cell viability was examined with the metabolic activity (MTT) assay, ROS generation was quantified via luminometry and the nitroblue-tetrazolium (NBT) test was applied to quantify the intracellular superoxide formation. Cell lysates were prepared at the end of the *in vitro* culture to investigate the intracellular activity of superoxide dismutase (SOD), catalase (CAT), glutathione peroxidase (GPx) as well as the concentrations of glutathione (GSH) and malondialdehyde (MDA).

**Results:**

FeAA treatment led to a reduced spermatozoa motility (*P* < 0.001), viability (*P* < 0.001) and a decline of the antioxidant capacity of spermatozoa (*P* < 0.001) but increased the ROS generation (*P* < 0.001), superoxide production (*P* < 0.001) and lipid peroxidation (*P* < 0.001). LYC administration resulted in a preservation of the spermatozoa motion parameters (*P* < 0.001), mitochondrial activity (*P* < 0.001) and antioxidant characteristics (*P* < 0.001 with respect to SOD; *P* < 0.01 in relation to CAT; *P* < 0.05 as for GPx and GSH) with a concentration range of 1 and 2 mmol/L LYC revealed to be the most effective.

**Conclusions:**

Our results suggest that LYC exhibits significant ROS-scavenging and antioxidant properties which may prevent spermatozoa alterations caused by oxidative stress, and preserve the functionality of male reproductive cells.

## Background

Oxidative stress (OS) is defined as a state of imbalance between reactive oxygen species (ROS) production and the ability to readily detoxify these reactive intermediates or to easily repair the resulting damage [1]. ROS are generated and needed during physiological processes related to spermatozoa structural and functional maturation [2] however pathologically increased ROS levels have been repeatedly associated with male reproductive dysfunction [3–5]. Spermatozoa are highly sensitive to OS as their plasma membranes are rich in polyunsaturated fatty acids - the principal target for oxidation [6], while their cytoplasm is mainly restricted to the midpiece with very few antioxidant mechanisms to provide adequate protection against oxidative damage [7]. Seminal OS may result in lipid peroxidation (LPO), DNA fragmentation, alterations to the cellular communication and enzymatic pathways [6, 7], which in turn are correlated with motility loss, alterations of membrane fusion events [6], poor fertilization rates or impaired embryogenesis [8, 9].

Over the past years numerous studies have shown that *in vitro* administration of hydrophilic or lipophilic antioxidants in human or veterinarian andrology may have positive effects on critical semen parameters including sperm motility, membrane and DNA integrity [10]. Moreover, antioxidants may protect spermatozoa from ROS produced by leukocytes, reduce cryodamage to spermatozoa, block premature sperm maturation and provide an overall stimulation to the male gamete [1, 11].

Lycopene (ψ, ψ-Carotene) (LYC) is a predominant natural carotenoid, which can be found in ripe tomato fruit, watermelon or pink grapefruit. Although used as a food colorant for many years, it has only recently become a subject of interest with respect to its properties in alleviating a numerous chronic or inflammatory diseases [12]. LYC is a highly unsaturated straight chain hydrocarbon with 13 double bonds, 11 of which are conjugated, which makes it a very powerful antioxidant. LYC has been shown to quench singlet oxygen twice as efficiently as β-carotene and ten times faster in comparison to α-tocopherol [13].

An increasing number of reports are emphasizing on the beneficial role of LYC supplementation in the management of reproductive dysfunction. Several human studies have shown that LYC administration leads to a significant improvement of semen parameters in patients diagnosed with idiopathic or antibody-mediated infertility [14, 15]. Moreover animal in vivo reports revealed that LYC may prevent testicular degeneration, improve sperm motility and morphology and stabilize the antioxidant profile of testicular tissue exposed to drugs [16], organic pollutants [17, 18] or mycotoxins [19].

Ferrous ascorbate has been shown to act as a highly suitable OS promoter to mammalian spermatozoa when these are deprived of the primary antioxidant protection provided by the seminal plasma [20–23]. Such system integrating ferrous and ascorbate ions reflects well on the chemistry and redox properties of iron, which as a transition metal has the ability to cause oxidative depletion of sperm lipids, proteins and DNA through the Fenton and Haber-Weiss reaction [6, 21, 24, 25].

Based on a pilot evidence stressing out a promising ability of LYC to provide antioxidant protection to male reproductive cells, this study was designed to explore the *in vitro* impact of LYC on bovine spermatozoa exposed to oxidative stress induced by ferrous ascorbate.

## Methods

### Experimental design

Ten adult Holstein Friesian breeding bulls (Slovak Biological Services, Nitra, Slovak Republic) were selected as semen donors for the scheduled experiments. One ejaculate was collected from each bull on a regular collection schedule (once a week for five consecutive weeks) using an artificial vagina. Immediately after collection, sperm concentration and motility was assessed using phase-contrast microscopy (200 ×). Only ejaculates with the required quality (minimum 70 % progressive motility and concentration of 1 × 10^9^ sperm/mL) were used for the subsequent experiments. All semen samples met the quality criteria given for the corresponding breed. By and large, 50 fresh ejaculates were used in the study. Institutional and national guidelines for the care and use of animals were followed, and all experimental procedures were approved by the State Veterinary and Food Institute of Slovak Republic (no. 3398/11-221/3) and Ethics Committee.

The *in vitro* treatment followed the protocol introduced by Bansal and Bilaspuri [21]. Each fresh semen sample was centrifuged (800 × g) at 25 °C for 5 min, seminal plasma was removed, the resulting pellet was washed twice with 2.9 % sodium citrate dissolved in distilled water (SC; pH 7.4; Centralchem, Bratislava, Slovak Republic), re-suspended in 2.9 % SC using a ratio of 1:20 (for cell lysis) or 1:40 (for immediate experimental assessments) and divided into ten equal fractions. To one fraction (Control 1; SC Control) only 2.9 % SC was added, and a different one (Control 2; FeAA Control) contained an OS inducer, i.e., ferrous ascorbate (FeAA) comprising 150 μmol/L FeSO_4_ (ferrous sulfate; FeSO_4_·7H_2_O; Sigma-Aldrich, St. Louis, MO, USA) and 750 μmol/L ascorbic acid (Centralchem), diluted in 2.9 % SC. The remaining eight (experimental) fractions were supplemented with 0.25, 0.5, 1 or 2 mmol/L lycopene dissolved in tetrahydrofuran (THF) containing 0.025 % butylated hydroxytoluene (BHT) (Sigma-Aldrich) in the presence or absence of FeAA (see Table 1). The final THF concentration was kept constant across all treatments (including both Controls) at a concentration of 0.1 %, chosen as having a minimal effect on cell viability. All suspensions were incubated at 37 °C.

**Table 1:** Concentrations of lycopene (LYC) used in the experiments

Spermatozoa fractions	LYC concentration, mmol/L
Fractions untreated with FeAA	
Fraction 1 (Control 1; SC Control)	0
Fraction 2	2
Fraction 3	1
Fraction 4	0.5
Fraction 5	0.25
Fractions treated with FeAA	
Fraction 6 (Control 2; FeAA Control)	0
Fraction 7	2
Fraction 8	1
Fraction 9	0.5
Fraction 10	0.25

At incubation periods of 0, 2 and 6 h spermatozoa motility parameters, ROS generation, cell viability and intracellular superoxide production were assessed in each fraction. Furthermore at 6 h each fraction was centrifuged at 800 × g at 25 °C for 10 min, the media were removed and the resulting pellet was sonicated at 28 kHz for 30 s on ice using RIPA buffer (Sigma-Aldrich) with protease inhibitor cocktail suitable for mammalian cell and tissue extracts (Sigma-Aldrich). Subsequently the samples were centrifuged at 11,828 × g, 4 °C for 15 min in order to purify the lysates from the residual cell debris. The resulting supernatants involving the intracellular content were stored at −80 °C for further assessment.

### Motility analysis

Spermatozoa motion parameters were assessed using the Computer-aided sperm analysis (CASA) system comprising the SpermVision™ program (Minitube, Tiefenbach, Germany) and Olympus BX 51 phase contrast microscope (Olympus, Tokyo, Japan). The system was set up as follows: frame rate - 60 Hz; minimum contrast - 20; static head size - 0.25 to 5.00; static head intensity - 0.40 to 2.00; static elongation - 20 to 100; default cell size - 4 pixels; default cell intensity - 40. Each sample was placed into the Makler Counting Chamber (depth 10 mm, 37 °C; Sefi Medical Instruments, Haifa, Israel) and at least 1000 cells were evaluated for motility (MOT; percentage of motile spermatozoa; motility >5 μm/s; %) and progressive motility (PROG; percentage of progressive motile spermatozoa; motility >20 μm/s; %).

### ROS generation

ROS production in each fraction was assessed by the chemiluminescence assay using luminol (5-amino-2, 3- dihydro-1, 4-phthalazinedione; Sigma-Aldrich) as the probe [26]. The test samples consisted of luminol (10 μL, 5 mmol/L) and 400 μL of control or experimental sample. Negative controls were prepared by replacing the sperm suspension with 400 μL of each culture medium. Positive controls included 400 μL of each medium, 10 μL luminol and 50 μL hydrogen peroxide (30 %; 8.8 M; Sigma-Aldrich). Chemiluminescence was measured on 48-well plates in 15 1 min-cycles using the Glomax Multi^+^ Combined Spectro-Fluoro Luminometer (Promega Corporation, Madison, WI, USA) [23]. The results are expressed as relative light units (RLU)/s/10^6^ sperm.

### Mitochondrial activity (MTT test)

Spermatozoa mitochondrial activity was evaluated using the colorimetric metabolic activity (MTT) test, which is based on the conversion of a yellow tetrazolium salt (3-(4,5-dimetylthiazol-2-yl)-2,5-diphenyltetrazolium bromide; MTT) to blue formazan particles by mitochondrial succinate dehydrogenase of intact mitochondria within living cells. The tetrazolium salt (Sigma-Aldrich) was dissolved in PBS (Dulbecco’s Phosphate Buffer Saline without calcium chloride and magnesium chloride; Sigma-Aldrich) at 5 mg/mL. Ten μL of the solution was added to each cell suspension. After a 2 h incubation (shaker, 37 °C, 95 % air atmosphere, 5 % CO_2_), the formazan crystals were dissolved in 80 μL of acidified (0.08 mol/L HCl; Centralchem) isopropanol (Centralchem). Optical density was determined at a wavelength of 570 nm against 620 nm as reference using a Multiskan FC microplate photometer (Thermo Fisher Scientific Inc., Waltham, MA, USA). Data are expressed as percentage of the SC Control (Control 1) set to 100 % [23].

### Quantification of the superoxide production (NBT test)

The nitroblue-tetrazolium (NBT) test was used to quantify the intracellular formation of the superoxide radical, by assessing blue NBT formazan deposits, generated by the reduction of the membrane permeable, yellow-colored, nitroblue tetrazolium chloride (2,20-bis(4-Nitrophenyl)-5,50-diphenyl-3,30-(3,30-dimethoxy-4,40-diphenylene) ditetrazolium chloride; Sigma-Aldrich) by the superoxide radical. The NBT salt was dissolved in PBS containing 1.5 % DMSO (dimethyl sulfoxide, Sigma-Aldrich) to a final concentration of 1 mg/mL and added to the cells (100 μL per well). After a 1 h incubation (shaker, 37 °C, 95 % air atmosphere, 5 % CO_2_), the cells were washed twice with PBS and centrifuged at 300 × g for 10 min. Lastly, the cells and formazan crystals were dissolved in 2 mol/L KOH (potassium hydroxide; Centralchem) in DMSO. Optical density was determined at a wavelength of 620 nm against 570 nm as reference by a Multiskan FC microplate photometer (Thermo Fisher Scientific Inc.). Data are expressed in percentage of the SC Control (Control 1) set to 100 % [23].

### Assessment of the antioxidant profile

Superoxide dismutase (SOD) activity was assessed using the Randox RANSOD commercial kit (Randox Laboratories, Crumlin, Great Britain) employing xanthine and xanthine oxidase (XO) to generate superoxide radicals, which will react with 2-(4-iodophenyl)-3-(4-nitrophenol)-5-phenyltetrazolium chloride (I.N.T.) to form a red formazan dye. SOD activity was subsequently measured by the inhibition degree of the reaction at 505 nm using the Genesys 10 spectrophotometer (Thermo Fisher Scientific Inc.). The results are expressed as U/mg protein.

Catalase (CAT) activity was quantified according to Beers and Sizer [27] by monitoring the decrease of hydrogen peroxide (H_2_O_2_) at 240 nm. The calculation was based on the rate of H_2_O_2_ decomposition, proportional to the reduction of the absorbance during 1 min measured with the Genesys 10 spectrophotometer. The values are expressed as U/mg protein.

Glutathione peroxidase (GPx) activity was evaluated using the Randox RANSEL commercial kit (Randox Laboratories), applying the method of Paglia and Valentine [28]. GPx catalyzes the oxidation of glutathione by cumene hydroperoxide. In the presence of glutathione reductase (Gr) and NADPH the oxidized glutathione is subsequently converted to the reduced form with a concomitant oxidation of NADPH to NADP^+^. The decrease of absorbance was measured using the Genesys 10 spectrophotometer (Thermo Fisher Scientific Inc.) at 340 nm. GPx activity is expressed as U/mg protein.

Reduced glutathione (GSH) was determined by the Ellman method [29]. Each sample was treated with DTNB (5,50-dithiobis-2-nitrobenzoic acid; Ellman’s reagent; Sigma-Aldrich) which interacts with the thiol groups of GSH, cleaving the disulfide bond to give 2-nitro-5-thiobenzoate (NTB^−^) and creating the NTB^2−^ dianion in water at alkaline pH. This ion has a yellow color and was quantified at 412 nm using the Genesys 10 spectrophotometer. GSH concentration is expressed as mg/g protein.

Lipid peroxidation (LPO) expressed through malondialdehyde (MDA) production was assessed with the help of the TBARS assay, modified for a 96-well plate and ELISA reader. Each sample was treated with 5 % sodium dodecyl sulfate (SDS; Sigma-Aldrich), and subjected to 0.53 % thiobarbituric acid (TBA; Sigma-Aldrich) dissolved in 20 % acetic acid adjusted with NaOH (Centralchem) to pH 3.5, and subsequently boiled at 90–100 °C for 1 h. Following boiling, the samples were placed on ice for 10 min and centrifuged at 1,750 × g for 10 min. Supernatant was used to measure the end-product resulting from the reaction of MDA and TBA under high temperature and acidic conditions at 530–540 nm with the help of the Multiskan FC microplate photometer (Thermo Fisher Scientific Inc.) [30]. MDA concentration is expressed as μmol/g protein.

Protein concentration was assessed using the DiaSys Total Protein (DiaSys, Holzheim, Germany) commercial kit and the semi-automated clinical chemistry photometric analyzer Microlab 300 (Merck, Darmstadt, Germany). The measurement is based on the Biuret method, according to which copper sulfate reacts with proteins to form a violet blue color complex in alkaline solution, and the intensity of the color is directly proportional to the protein concentration when measured at 540 nm.

### Statistical analysis

Statistical analysis was carried out using the GraphPad Prism program (version 3.02 for Windows; GraphPad Software, La Jolla, CA, USA, http://www.graphpad.com). Descriptive statistical characteristics (mean, standard error) were evaluated at first. One-way ANOVA was used for specific statistical evaluations. Dunnett’s test was applied as a follow-up test to ANOVA, based on a comparison of every mean to a control mean, and computing a confidence interval for the difference between the two means. The level of significance was set at 0.05, and *** means *P* < 0.001, ** means *P* < 0.01, * means *P* < 0.05. The comparative analysis was performed as follows:

1. SC Control (Control 1) was compared to the FeAA Control (Control 2)
2. Experimental fractions not subjected FeAA treatment were compared to the SC Control exclusively (Control 1)
3. Experimental fractions subjected to FeAA treatment were compared to the SC Control (Control 1) as well as to the FeAA Control (Control 2).

## Results

The CASA analysis revealed a significant (*P* < 0.001) decrease of both motion characteristics over the course of the *in vitro* incubation as a consequence of FeAA administration (Table 2). Supplementation of 0.5–2 mmol/L LYC to the experimental fractions untreated with FeAA resulted in a significantly increased MOT and PROG in comparison with the Control 1 at 2 h (*P* < 0.05) as well as 6 h (*P* < 0.001 in case of 1–2 mmol/L LYC; *P* < 0.01 with respect to 0.5 mmol/L LYC; MOT; Table 2). Furthermore, 0.5–2 mmol/L LYC administration to the FeAA fractions led to a significant improvement of both motion parameters (*P* < 0.001; Times 2 h and 6 h) when compared to the Control 2 (FeAA Control), although none of the selected LYC concentrations was able to entirely reverse the negative impact of FeAA on the sperm motility parameters (Table 2).

**Table 2:** Spermatozoa motility parameters affected by four doses of lycopene (LYC), untreated vs. treated with ferrous ascorbate (FeAA)

Fractions										
Fractions untreated with FeAA						Fractions treated with FeAA				
	Ctrl 1 (SC Ctrl)	2 mmol/L LYC	1 mmol/L LYC	0.5 mmol/L LYC	0.25 mmol/L LYC	Ctrl 2 (FeAA Ctrl)	2 mmol/L LYC	1 mmol/L LYC	0.5 mmol/L LYC	0.25 mmol/L LYC
Time 0 h										
MOT, %	94.23 ± 0.50	95.76 ± 0.40	95.67 ± 0.56	95.24 ± 0.58	94.70 ± 0.59	92.31 ± 0.76	94.53 ± 0.58	93.40 ± 2.42	93.83 ± 0.76	93.20 ± 0.56
PROG, %	84.26 ± 0.77	87.45 ± 0.57	86.23 ± 0.73	85.10 ± 1.09	85.66 ± 0.83	82.90 ± 1.20	85.14 ± 0.75	84.52 ± 2.29	83.64 ± 1.01	83.93 ± 0.83
Time 2 h										
MOT, %	75.71 ± 1.08	86.62 ± 2.78*^1^	85.65 ± 2.19*^1^	85.01 ± 1.31*^1^	82.30 ± 1.89	61.32 ± 1.54***^1^	80.58 ± 1.63***^2^	75.61 ± 1.95***^2^	73.57 ± 2.28***^2^	63.21 ± 1.76***^1^
PROG, %	67.52 ± 1.62	79.43 ± 3.28*^1^	79.40 ± 2.74^*1^	78.05 ± 2.22*^1^	75.31 ± 2.91	48.22 ± 2.75***^1^	62.71 ± 2.28***^2^	61.93 ± 2.76***^2^	58.08 ± 1.14	49.55 ± 1.04***^1^
Time 6 h										
MOT, %	53.59 ± 2.50	69.02 ± 2.72***^1^	67.79 ± 1.96***^1^	65.43 ± 2.49*^1^	54.72 ± 2.69	25.18 ± 2.94***^1^	44.21 ± 2.94***^2^	42.10 ± 2.95*^1;^ ***^2^	41.71 ± 2.45*^1;^ ***^2^	37.56 ± 1.78*^1;^ *^2^
PROG, %	42.61 ± 2.22	56.60 ± 2.91**^1^	55.05 ± 1.85**^1^	53.72 ± 2.75*^1^	52.57 ± 2.53*^1^	20.38 ± 1.17***^1^	44.62 ± 1.54***^2^	39.03 ± 2.00***^2^	29.83 ± 2.79*^2^	25.89 ± 1.26***^2^

Mean ± Standard Error

*MOT* spermatozoa motility, *PROG* spermatozoa progressive motility

*P < 0.05; **P < 0.01; ***P < 0.001

^1^ – vs. Control 1 (SC Control)

^2^ – vs. Control 2 (FeAA Control)

**Fig. 1:**
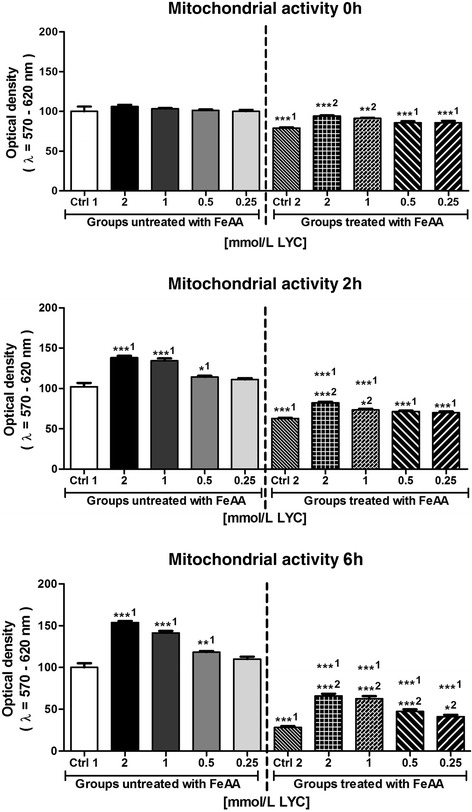
Mitochondrial activity of bovine spermatozoa affected by four doses of lycopene (LYC), untreated or co-treated with ferrous ascorbate (FeAA). Each bar represents mean (±SEM) optical density as the percentage of the Control 1 (SC control), which was set to 100 % and the data are expressed as a % of the Control 1 value. The data were obtained from five independent experiments. The level of significance was set at 0.05, and *** means *P* < 0.001, ** means *P* < 0.01, * means *P* < 0.05. ^1^ – vs. Control 1, ^2^ – vs. Control 2

Consistently with the decreased motion parameters, a decrease of spermatozoa mitochondrial activity was recorded after FeAA administration, with significant differences at all timeframes of the *in vitro* culture (*P* < 0.001; Fig. 1). 0.5–2 mmol/L LYC supplemented to the FeAA untreated samples exhibited a significant activity-promoting effect on the sperm viability (*P* < 0.01 with respect to 1 and 2 mmol/L LYC; Times 2 h and 6 h). At the same time, 1 and 2 mmol/L LYC exhibited the capacity to at least partially prevent the decline of mitochondrial activity in the fractions subjected to FeAA treatment immediately after the *in vitro* culture had started (*P* < 0.01 in case of 1 mmol/L LYC; *P* < 0.001 with respect to 2 mmol/L LYC; Time 0 h), maintaining their protective effects to the end of the experiment (*P* < 0.05 given 0.25 mmol/L LYC; *P* < 0.001 in case of 0.5–2 mmol/L LYC; Time 6 h; Fig. 1).

**Fig. 2:**
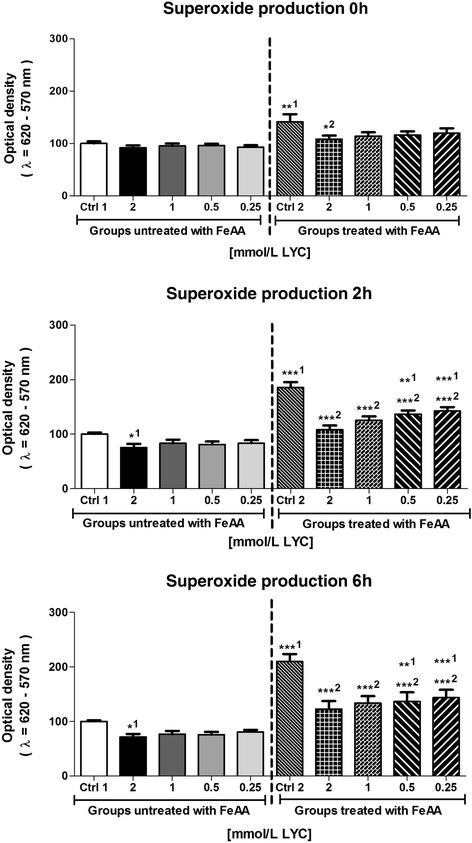
Intracellular superoxide production of bovine spermatozoa affected by four doses of lycopene (LYC), untreated or co-treated with ferrous ascorbate (FeAA). Each bar represents mean (±SEM) optical density as the percentage of the Control 1 (SC control), which was set to 100 % and the data are expressed as a % of the Control 1 value. The data were obtained from five independent experiments. The level of significance was set at 0.05, and *** means *P* < 0.001, ** means *P* < 0.01, * means *P* < 0.05. ^1^ – vs. Control 1, ^2^ – vs. Control 2

The decrease of motility and viability of bovine spermatozoa in the FeAA Control was accompanied by an increase in the ROS generation as well as superoxide production (Table 3; Fig. 2). Compared to the Control 1, ROS and superoxide overproduction significantly increased (*P* < 0.01) practically the moment FeAA was added to the sperm suspension (Time 0 h), followed by a further rise of their intensity over the time of the *in vitro* culture (*P* < 0.001). On the other hand, LYC administration led to a decrease in the ROS as well as superoxide generation when compared to the Control 1, with significant effects particularly in case of 2 mmol/L LYC (*P* < 0.01 with respect to ROS; *P* < 0.05 in relation to NBT). LYC supplementation to the FeAA-treated fractions did not completely reverse the free radical overproduction, however it was able to significantly decrease both ROS and superoxide concentration when compared to the FeAA Control (*P* < 0.001 with respect to 1–2 mmol/L LYC; Table 3; Fig. 2).

**Table 3:** Reactive oxygen species (ROS) production by bovine spermatozoa [RLU/s/10^6^ sperm] affected by four doses of lycopene (LYC), untreated vs. treated with ferrous ascorbate (FeAA)

Fractions									
Fractions untreated with FeAA					Fractions treated with FeAA				
Ctrl 1 (SC Ctrl)	2 mmol/L LYC	1 mmol/L LYC	0.5 mmol/L LYC	0.25 mmol/L LYC	Ctrl 2 (FeAA Ctrl)	2 mmol/L LYC	1 mmol/L LYC	0.5 mmol/L LYC	0.25 mmol/L LYC
Time 0 h									
1.63 ± 0.32	1.01 ± 0.22	1.21 ± 0.30	1.30 ± 0.16	1.57 ± 0.30	4.22 ± 0.70**^1^	2.00 ± 0.49*^2^	2.12 ± 0.41*^2^	2.55 ± 0.39	3.16 ± 0.58*^1^
Time 2 h									
4.49 ± 0.42	2.49 ± 0.72	2.58 ± 0.50	3.66 ± 0.19	4.10 ± 0.25	9.75 ± 0.85***^1^	5.28 ± 0.22**^2^	5.79 ± 0.45**^2^	7.90 ± 0.48*^1^	8.11 ± 0.65**^1^
Time 6 h									
10.92 ± 0.62	6.55 ± 0.21**^1^	7.01 ± 0.54**^1^	8.66 ± 0.52	9.15 ± 0.36	23.55 ± 1.51***^1^	12.33 ± 1.09***^2^	14.77 ± 0.80***^2^	17.00 ± 1.21*^2,^ **^1^	20.25 ± 1.56***^1^

Mean ± Standard Error

*P < 0.05; **P < 0.01; ***P < 0.001

^1^ – vs. Control 1 (SC Control)

^2^ – vs. Control 2 (FeAA Control)

Assessment of the antioxidant profile revealed that FeAA treatment led to a significant decrease of SOD, CAT and GPx activities (*P* < 0.01 with respect to SOD; *P* < 0.001 in relation with CAT and GPx) as well as a decline of the GSH concentration (*P* < 0.05; Table 4). Inversely, a significant increase of the MDA concentration (*P* < 0.001) was detected following FeAA treatment (Table 4). Evaluating the impact of LYC on the intracellular antioxidant molecules under physiological conditions it was revealed that 1 and 2 mmol/L LYC were able to stimulate the GPx activity as compared to the Control 1 (*P* < 0.05). A significant association between LYC supplementation and changes in the activities of the enzymatic antioxidants were recorded in the FeAA experimental fractions exposed to 1 and 2 mmol/L LYC (*P* < 0.01 with respect to SOD; *P* < 0.001 in case of CAT; *P* < 0.05 in relation to GPx). Similarly, a significant increase in the GSH concentration was recorded in the FeAA-supplemented experimental fractions following the addition of 1 and 2 mmol/L LYC (*P* < 0.05; Table 3). All LYC concentrations exhibited lipoprotective effects by preventing MDA overgeneration when compared to the FeAA Control (*P* < 0.001; Table 4).

**Table 4:** Markers of oxidative balance in bovine spermatozoa affected by four doses of lycopene (LYC), untreated vs. treated with ferrous ascorbate (FeAA)

Fractions									
Fractions untreated with FeAA					Fractions treated with FeAA				
Ctrl 1 (SC Ctrl)	2 mmol/L LYC	1 mmol/L LYC	0.5 mmol/L LYC	0.25 mmol/L LYC	Ctrl 2 (FeAA Ctrl)	2 mmol/L LYC	1 mmol/L LYC	0.5 mmol/L LYC	0.25 mmol/L LYC
SOD, U/mg prot									
0.43 ± 0.06	0.47 ± 0.02	0.45 ± 0.06	0.45 ± 0.07	0.44 ± 0.04	0.31 ± 0.05**^1^	0.43 ± 0.06^**2^	0.42 ± 0.07**^2^	0.39 ± 0.01	0.39 ± 0.01
CAT, U/mg prot									
9.14 ± 0.86	11.75 ± 0.78	10.06 ± 1.05	10.68 ± 1.08	9.72 ± 1.04	4.85 ± 0.71***^1^	9.83 ± 0.57***^2^	9.68 ± 1.25***^2^	8.54 ± 0.58**^2^	8.23 ± 0.72
GPx, U/mg prot									
0.088 ± 0.016	0.121 ± 0.019*^1^	0.119 ± 0.019*^1^	0.099 ± 0.006	0.090 ± 0.039	0.037 ± 0.010***^1^	0.066 ± 0.003^*2^	0.066 ± 0.016*^2^	0.058 ± 0.021	0.057 ± 0.004
GSH, mg/g prot									
8.10 ± 1.02	11.14 ± 1.21	8.35 ± 1.80	8.87 ± 1.10	8.27 ± 1.15	5.51 ± 0.99*^1^	7.53 ± 0.84*^2^	7.53 ± 1.24*^2^	7.21 ± 0.69	6.72 ± 0.97
MDA, μmol/g prot									
4.27 ± 0.24	3.09 ± 0.26	3.32 ± 0.33	3.33 ± 0.38	3.65 ± 0.38	10.25 ± 0.75***^1^	4.21 ± 0.25***^2^	4.35 ± 0.24***^2^	4.52 ± 0.14***^2^	4.73 ± 0.40***^2^

Mean ± Standard Error

*SOD* superoxide dismutase activity, *CAT* catalase activity, *GPx* glutathione peroxidase activity, *GSH* concentration of reduced glutathione, *MDA* malondialdehyde concentration

*P < 0.05; **P < 0.01; ***P < 0.001

^1^ – vs. Control 1 (SC Control)

^2^ – vs. Control 2 (FeAA Control)

## Discussion

It has been emphasized that ferrous ascorbate is a highly efficient OS-promoting system in mammalian spermatozoa [6, 25] by providing a suitable environment for the Fenton and Haber-Weiss reaction [31]. This molecule reflects well on the specific properties of numerous transition metals including copper, iron, zinc or manganese. These essential micronutrients participate in the control of diverse metabolic and signalling pathways. On the other hand, their rich coordination chemistry and redox properties allow them to cause oxidative deterioration of sperm DNA, lipids and depletion of protein sulfhydryls [6, 25]. Such alterations to essential biomolecules are in turn associated with a reduction in motility and mitochondrial metabolism of spermatozoa, which can lead to the inactivation of enzymes involved in glycolysis and oxidative phosphorylation [20, 32–34].

Correspondingly to our CASA analysis, a significant decline in the spermatozoa motion parameters accompanied by an increased ROS generation following exposure to FeAA has been reported on human [6], bovine [21, 23], murine [22], deer [25] and equine [35] spermatozoa. Interestingly, Baumber et al. [35] reported that although greater ROS concentrations were associated with a decreased spermatozoa motility in stallions as a result of FeAA administration, no changes were observed in the sperm viability, mitochondrial activity or acrosomal integrity.

To assess if FeAA had any impact on the intracellular antioxidant activity of spermatozoa, this study was conducted to assess the activity of antioxidant enzymes, hypothesized to be the first line of defense against OS [36]. SOD, CAT as well as GPx activities were significantly decreased following FeAA administration, revealing a severe inability of the enzymatic antioxidant system to readily detoxify inracellular ROS, and to protect the sperm cellular structures against oxidative insults. A similar depletion of antioxidant enzymes was previously reported by Mojica-Villegas et al. [22] and Tvrdá et al. [23]. As opposed to enzymatic antioxidants, GSH has been reported to decrease in concentration as a response to OS in some studies [23, 37] while in other studies an increased GSH amount has been observed following ROS overgeneration [38]. A decrease of GSH resulting from FeAA administration in this study may be a result of possible oxidative damage to the sulphydryl groups of GSH, responsible for ROS trapping.

Lipid peroxidation is well known to be the primary mechanism of oxidative damage to spermatozoa [6]. This process may lead to alterations in the membrane integrity, followed by increased concentrations of lipid hydroperoxides, alkoxyl and/or peroxyl radicals, and resulting in the production of cytotoxic aldehydes including MDA [6, 36] in accordance with the present data as well as with previous studies where mammalian spermatozoa were exposed to FeAA [21–24, 35].

To protect male reproductive cells from the deleterious effects of free radicals, numerous clinical and experimental trials using antioxidant agents have been attempted. Carotenoids, as potential antioxidants, are known to be highly efficient scavengers of singlet oxygen (^1^O_2_) and other ROS. During ^1^O_2_ quenching, energy is transfered from ^1^O_2_ to the LYC molecule, converting it to the energy-rich triplet state. Trapping other ROS, such as the hydroxyl radical (**·**OH), nitrogen dioxide (**·**NO_2_) or peroxynitrite, in contrast, may lead to oxidative breakdown of LYC [16, 39]. As such, LYC may offer protection against oxidation of lipids, proteins, and DNA [14, 40, 41]. LYC has been shown to have the highest antioxidant activity among carotenoids with respect to cell protection against H_2_O_2_ and **·**NO_2_. In addition, LYC has been reported to attenuate OS and reverse testicular toxicopathology both *in vitro* and in vivo [16, 39, 42, 43].

Previous reports on the role of LYC in male reproduction have to a large extent shown that this molecule may exhibit a significant protective effect on the sperm activity and oxidative balance. Nevertheless we must bear in mind, that whole ejaculates and/or extenders were used, therefore the beneficial effects LYC exhibited on the sperm survival could have been caused by a synergy between LYC and a broad array of antioxidants or protective molecules found in the seminal plasma and/or extenders. As such, the present study aimed to validate if LYC has the ability to independently contribute to the spermatozoa protection against metal-induced oxidative damage.

Our experiments show that LYC administration significantly improved sperm motility parameters under both physiological and oxidative *in vitro* conditions. Contrary to our *in vitro* study, Mangiagalli et al. [44] reported that in vivo administration of 0.1 or 0.5 g/L LYC had no significant impact on the motility rate or forward progressive motility in fresh rabbit semen. Although in vivo LYC supplementation showed not to be associated with an improvement of male reproductive performance in rabbits, Gupta and Kumar [14] as well as Eskenazi et al. [45] examining human subjects reported that a higher LYC intake was associated with a greater sperm concentration and motility.

*In vitro* protective properties of LYC on the sperm survival were reported in chilled fowl and bull cryopreserved semen [46, 47]. Correspondingly to our observations the authors noted that LYC supplementation led to a significantly increased sperm motility and viability after *in vitro* storage, as a result of specific protective effects of this molecule against cell damage, probably through its ROS-quenching abilities and prevention of LPO.

LYC supplementation in our experiments had a dose-dependent positive effect especially in preventing the decrease of spermatozoa motion and mitochondrial activity. Similar positive outcomes of LYC administration were reported by Mangiagalli et al. [44] in case of rabbit sperm motility and viability in samples stored for 24 h at 5 °C. Similarly to our observations Uysal and Bucak [48] noted that LYC supplementation to a culture medium for ram semen prevented typical deleterious effects of semen storage on spermatological indicators such as a decline in sperm motility and increased sperm abnormalities, acrosome damage or dead sperm.

Beneficial effects of LYC supplementation related to the prevention of ROS overgeneration and stabilization of the sperm antioxidant profile found in our trial complement reports on the alleviating role of LYC on the structure or function of the male reproductive system in animal and human subjects. Türk et al. [49] reported that LYC administration in rats treated with cyclosporine A (CsA) significantly increased the sperm concentration, motility and decreased ROS generation in comparison to the CsA-treated control, confirming the role of LYC as a potential protective agent against structural and functional damage to the male reproductive cell. Ateşşahin et al. [39] found that the presence of LYC significantly improved the semen quality and antioxidant capacity in rats treated with cisplatin due to its ability to reverse ROS production and oxidative damage. According to Zini et al. [50] preincubation of human spermatozoa with LYC caused a significantly lower DNA damage of male reproductive cells subjected to hydrogen peroxide. On the other hand, no improvement of sperm motion parameters was recorded in this case.

The outcomes of this study show that LYC has the ability to modulate the antioxidant profile of male gametes. Similar conclusions were drawn by Tamiselvan et al. [18], Türk et al. [49] and Salem et al. [51] reporting that LYC administration resulted in a normalization of the antioxidant status together with a stabilization of SOD, CAT, GPx and GSH followed by a decrease of H_2_O_2_ production and MDA synthesis in male reproductive cells and tissues.

According to Aly et al. [52], LYC supplemented before lipopolysaccharide (LPS) treatment attenuated the mitochondrial damage in male germ cells. Protective effects of LYC were accompanied by a decrease of MDA and H_2_O_2_ generation, suggesting a ROS-trapping ability of this carotene. Moreover LYC treatment prevented the decrease of SOD, CAT, GPx and Gr activities, normalized GSH and vitamin C concentrations which subsequently contributed to the ROS-scavenging activities of the system.

In this study, LYC significantly inhibited the increase of MDA in the sperm suspensions as a possible consequence of its ability to interreact with oxygen metabolites before these can reach and oxidize lipid biomolecules. A similar MDA decrease has been reported by Filipcikova et al. [15] emphasizing on the efficacy of LYC in changing PUFA levels in the seminal of previously infertile subjects. Taş et al. [19] speculate that this LPO-preventing potential lies in the ability of LYC to become entrapped in the hydrophobic core of membranous constituents in spermatozoa. Moreover Goyal et al. [53] have previously presented evidence on how LYC accumulates in lipid-rich seminal prostasomes, providing them protection against degradation. Further studies using prostatic epithelial cells also confirmed LYC packaging into analogous microparticles before secretion from the cell [54].

Positive effects of LYC on either improving or restoring male fertilitzation may be based on two hypotheses. One explanation for our results is that LYC is a lipophilic substance which easily passes through biological membranes and rapidly enters into the cell. A second possible mechanism is that LYC plays an important role in the protection of cellular membranes and lipoproteins against oxidative damage [55]. It is possible that the provitamin A activity of β-carotene has a direct effective role in this protective mechanisms. Compatible to our results, β-carotene has been reported to prevent LPO and attenuate the decline of antioxidant enzymes in male reproductive tissues [56].

## Conclusions

In conclusion, LYC was capable of preventing the decline of spermatozoa vitality, functional activity and antioxidant capacity as a consequence of FeAA-associated oxidative damage. LYC concentrations ranging between 1 and 2 mmol/L were particularly effective in protecting the male reproductive cell against alterations caused by ROS overgeneration through prevention of LPO and stabilization of the antioxidant profile, translated into the maintenance of sperm motility and mitochondrial activity. Correspondingly, LYC supplementation may be a suitable strategy to preserve the vitality of male reproductive cells and to cease oxidative insults to the sperm structural integrity and functional activity.
